# Wilms tumour resulting from paternal transmission of a *TRIM28* pathogenic variant—A first report

**DOI:** 10.1038/s41431-024-01545-7

**Published:** 2024-01-29

**Authors:** James Whitworth, Ruth Armstrong, Eamonn R. Maher

**Affiliations:** 1 University of Cambridge Department of Medical Genetics, Box 238 Level 6, Addenbrooke’s Treatment Centre, Cambridge University Hospitals NHS Foundation Trust, Hills Road, Cambridge, CB2 0QQ UK; 2 Department of Clinical Genetics, Box 134 Level 6, Addenbrooke’s Treatment Centre, Cambridge University Hospitals NHS Foundation Trust, Hills Road, Cambridge, CB2 0QQ UK; 3 Aston Medical School, Aston University, Birmingham, B4 7ET UK

**Keywords:** Embryonal neoplasms, Paediatrics

## Abstract

Wilms tumour (nephroblastoma) is a renal embryonal tumour that is frequently caused by constitutional variants in a small range of cancer predisposition genes. *TRIM28* has recently been identified as one such gene. Previously, observational data strongly suggested a parent of origin effect, whereby Wilms tumour only occurred following maternal inheritance of a pathogenic genetic variant. However, here we report a child with bilateral Wilms tumour who had inherited a pathogenic *TRIM28* variant from their father. This finding suggests that genetic counselling for paternally inherited pathogenic variants in *TRIM28* should include discussion of a potential risk of Wilms tumour.

## Introduction

Wilms tumour (nephroblastoma) is an embryonal tumour of kidney tissue that is the most frequent renal neoplasm in childhood. It results from abnormal nephrogenesis and is associated with aberrant mesenchymal to epithelial transition [1]. Most Wilms tumours occur as isolated cases and recent evidence has demonstrated that in some instances, post-zygotic genomic events early in embryogenesis lead to pre-malignant clonal expansions that are widespread in kidney tissue from affected individuals [2].

Additionally, there are a number of inherited causes that occur due to constitutional pathogenic variants (e.g. in *WT1, DIS3L2, REST, BUB1B* etc) [3] and Wilms tumour predisposition is a well-recognised feature of the congenital imprinting disorder Beckwith-Wiedemann Syndrome [4]. Recently, *TRIM28* has been described independently as a further Wilms predisposition gene by multiple groups and appears particularly associated with the rarer epithelial histological subtype along with loss of the wild type allele in tumour [5–11]. *TRIM28* is located in the terminal part of the long arm of chromosome 19 and encodes a transcriptional co-repressor. It has been implicated in a variety of cellular processes that are potentially relevant to Wilms tumour including epithelial to mesenchymal transition, DNA damage response, stem cell maintenance [12], and kidney development [13].

A striking feature of reported Wilms tumour cases associated with constitutional *TRIM28* variants is an apparent parent of origin effect whereby the neoplasm only manifests following maternal inheritance of the genetic variant. Such parent-of-origin effects are a characteristic of pathogenic variants in genes which undergo genomic imprinting (i.e. are expressed from only one parental allele in a parent-specific manner) [14]. However, parent-of-origin effects on tumour risks have also been described in other tumour predisposition syndromes caused by variants in non-imprinted genes (e.g. phaeochromocytoma/paraganglioma (PPGL) due to constitutional *SDHD* and *MAX* variants) when the predisposition gene maps to a chromosome (e.g. 11 and 14) that contain an imprinted gene cluster [15, 16]. Here, we report the first case of Wilms tumour due to documented paternal transmission of a *TRIM28* pathogenic variant.

## Subjects and methods

The family was ascertained via referral to the Department of Clinical Genetics, Cambridge University Hospitals. Previous research conducted by the Institute of Cancer Research as part of the Factors Associated with Childhood Tumours (FACT) study had identified the *TRIM28* variant, which was confirmed in a diagnostic laboratory. Consent for further study was through participation in the (Molecular Pathology of Human Genetic Disease study) and written informed consent for publication was obtained. Molecular analysis of tumour tissue was attempted by preparing sections from formalin fixed paraffin embedded (FFPE) biopsy samples from the left and right kidneys. However, DNA was not of sufficient quality for reliable sequencing.

## Results

### Case report

A female proband presented with abdominal distension and hypertension at the age of nine months and was diagnosed with bilateral Wilms tumour (with two masses on the left), which was treated with chemotherapy and nephron sparing surgery. Histological examination of both left and right tumour biopsies showed predominantly epithelial structures and some blastemal areas with positive immunostaining for WT-1, INI-1 and CD56. Nephrogenic rests were considered likely to be present.

There was no known family history of cancer other than a lymphoma diagnosis in the paternal grandfather.

### Molecular studies

Genetic investigation of blood DNA with chromosomal microarray and *WT1* testing did not demonstrate any potentially causative variants and the patient was recruited to a research study for further consideration of a genetic cause. This demonstrated a constitutional *TRIM28* nonsense variant (NM_005762 c.688 C > T p.(Arg230Ter)) via exome sequencing that was deemed pathogenic and causative of the Wilms tumours. Subsequent testing of parental samples for the variant showed that it had been unexpectedly inherited from the father, who had no significant medical history at age 36 years. No fertility treatment was known to have been necessary for the pregnancy and the couple have two other children. No other Wilms tumours had occurred in the family.

The proband continued under oncology follow up with ultrasound scans every three months until age seven years with six monthly scans thereafter. Three monthly scans were recommended until age seven years for other carriers of the paternally inherited variant in the family. Individuals with the variant who were above the age for Wilms tumour surveillance [3] had a single one-off scan, which showed two likely renal angiomyolipomas in the case of the father but no other abnormalities.

## Discussion

The assertion that only maternal inheritance of *TRIM28* variants predisposes to Wilms tumour arises from the observation that in one of the originally described series, 10/10 inherited (as opposed to de novo) variants were also present in the mother [5] and no instances of paternal inheritance were noted in other reports [6, 8, 9]. Such parent of origin effects in tumour predisposition syndromes have been described previously, notably for *SDHD, SDHAF2* and *MAX* pathogenic variants, which are associated with PPGL when paternally inherited [15, 16].

Perhaps the most intuitive molecular explanation for parent of origin effects is that the allele from one parent is inactive in the normal state due to an imprint established during gametogenesis. In that scenario, inheritance of a pathogenic loss of function variant from that parent would not cause additional aberration of gene function but inheritance from the other parent would lead to two non-functional alleles. However, to date no evidence has been reported that *TRIM28* is an imprinted gene [17, 18].

**Fig. 1:**
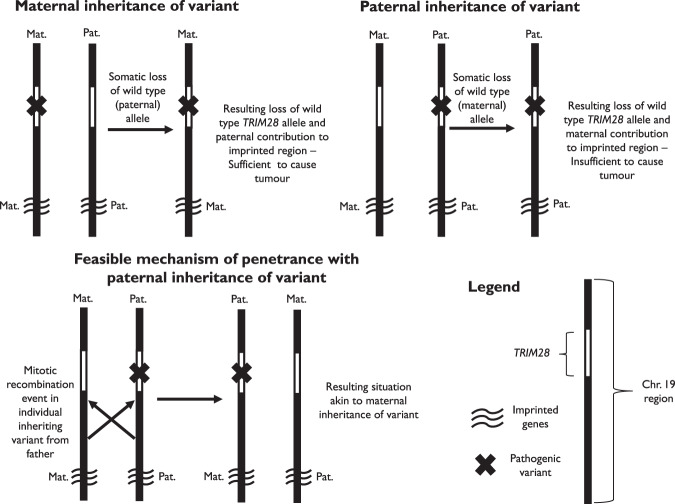
Potential mechanism of penetrance resulting from maternal inheritance of *TRIM28* variants and paternal inheritance in exceptional cases.

An alternative explanation of parent-of-origin effects associated with a non-imprinted cancer predisposition gene is that proposed for PPGL associated with pathogenic variants in *SDHD* and *SDHAF2,* which both map to chromosome 11 and predispose to PPGL when pathogenic variants are paternally inherited. In these cases, PPGL usually show loss of the maternally inherited chromosome 11 in the tumour tissue. This results in loss of the maternally expressed imprinted gene *CDKN1C* whilst the function of the paternally expressed growth factor *IGF2* is unaffected [19]. In contrast when a germline *SDHD*/*SDHAF2* variant is maternally inherited, though somatic loss of the paternal chromosome 11 would be associated with biallelic *SDHD* inactivation, there would also be loss of the functioning paternal *IGF2* allele but the functional *CDKN1C* allele would be unaffected. Based on studies of PPGL associated with pathogenic variants in *SDHD*, it would be predicted that *TRIM28* tumourigenesis would be favoured by somatic events that resulted in biallelic *TRIM28* inactivation accompanied by loss of paternally expressed imprinted genes (and/or preservation of maternally expressed imprinted genes) mapping to chromosome 19. Penetrance from paternally inherited variants under this model would be due to mitotic recombination events involving this region (Fig. 1). Whilst poor DNA quality precluded investigation of FFPE- tumour material in the present case, other groups have found that in Wilms tumours associated with constitutional or somatic *TRIM28* pathogenic variants, loss of heterozygosity (LOH) can involve imprinted genes on chromosome 19, albeit without complete consistency in terms of included genes. Halliday et al. reported LOH in a Wilms tumour from an individual with a constitutional *TRIM28* frameshift variant where a distal q13.43 region of homozygosity included eight genes [6], none of which are firmly considered as imprinted [17]. Armstrong et al. performed copy number analysis in five Wilms tumours with somatic *TRIM28* mutations, four of which showed copy number neutral LOH at 19q13.32 to 19q13.43 [7] that includes *TRIM28* along with a number of reported paternally expressed imprinted genes (*ZIM2*, *PEG3*, *MIMT1*, *MIR371A*) [17, 18]. If the Halliday and Armstrong studies are taken together, no known imprinted genes are within the region of LOH in all tumours although some affected in multiple samples have greater potential relevance such as *PEG3*, a paternally expressed gene that was hypothesised as of potential relevance by Mahamdallie et al. [5]. It is a regulator of the tumour necrosis factor immune response and decreased expression has been associated with tumourigenesis [20].

Another mechanism that has been proposed to explain the parent-of-origin effect is that *TRIM28* pathogenic variants could affect spermatogenesis and lead to decreased male fertility. This has been supported by the observation of testicular degeneration and premature infertility in heterozygous *TRIM28* knockout mice [21] in addition to some reported male pathogenic variant carriers not having fathered children [5, 8]. However, there was no indication of subfertility in the male carrier in this case.

This report of Wilms tumour in the context of a paternally inherited *TRIM28* pathogenic variant demonstrates that this mode of inheritance is possible despite pre-existing evidence of a parent of origin effect where penetrance would only result from maternal inheritance. The mechanistic basis of this observation is unclear but more frequent paired WGS analysis of Wilms tumours in clinical practice may beget the definition of common regions of LOH and mitotic recombination events. Although influenced by ascertainment bias, the penetrance of maternally inherited *TRIM28* pathogenic variants has been estimated at around 67% [5, 8, 9]. Whilst identification of a paternally inherited *TRIM28* variant in a child appears to confer a lower risk, we suggest that clinicians still consider surveillance for Wilms tumour, particularly if it has been penetrant in a sibling. The question of surveillance is likely to arise with increasing frequency in the UK given that *TRIM28* is one of only five cancer predisposition genes included in the Genomics England Newborn Genomes Programme [22].

## Data Availability

Data sharing not applicable to this article as no datasets were generated or analysed during the current study.
